# A summary of eight traits of Coleoptera, Hemiptera, Orthoptera and Araneae, occurring in grasslands in Germany

**DOI:** 10.1038/sdata.2015.13

**Published:** 2015-03-31

**Authors:** Martin M Gossner, Nadja K Simons, Roland Achtziger, Theo Blick, Wolfgang H.O Dorow, Frank Dziock, Frank Köhler, Wolfgang Rabitsch, Wolfgang W Weisser

**Affiliations:** 1 Terrestrial Ecology Research Group, Department of Ecology and Ecosystem Management, Center for Life and Food Sciences Weihenstephan, Technische Universität München, Hans-Carl-von-Carlowitz-Platz 2, D-85354 Freising, Germany; 2 TU Bergakademie Freiberg, Institute for Biosciences, Biology & Ecology Unit, Leipziger Straße 29, D-09599 Freiberg, Germany; 3 Callistus—Gemeinschaft für Zoologische & Ökologische Untersuchungen, Heidloh 8, D-95503 Hummeltal, Germany; 4 Senckenberg Research Institute and Natural History Museum, Senckenberganlage 25, D-60325 Frankfurt am Main, Germany; 5 Faculty of Agriculture/Landscape Management, HTW Dresden University of Applied Sciences, Pillnitzer Platz 2, D-01326 Dresden, Germany; 6 Strombergstr. 22a, D-53332 Bornheim, Germany; 7 Environment Agency Austria, Dept. Biodiversity & Nature Conservation, Spittelauer Lände 5, A-1090 Vienna, Austria

**Keywords:** Community ecology, Biodiversity, Entomology, Grassland ecology

## Abstract

Analyses of species traits have increased our understanding of how environmental drivers such as disturbances affect the composition of arthropod communities and related processes. There are, however, few studies on which traits in the arthropod community are affected by environmental changes and which traits affect ecosystem functioning. The assembly of arthropod traits of several taxa is difficult because of the large number of species, limited availability of trait databases and differences in available traits. We sampled arthropod species data from a total of 150 managed grassland plots in three regions of Germany. These plots represent the spectrum from extensively used pastures to mown pastures to intensively managed and fertilized meadows. In this paper, we summarize information on body size, dispersal ability, feeding guild and specialization (within herbivores), feeding mode, feeding tissue (within herbivorous suckers), plant part (within herbivorous chewers), endophagous lifestyle (within herbivores), and vertical stratum use for 1,230 species of Coleoptera, Hemiptera (Heteroptera, Auchenorrhyncha), Orthoptera (Saltatoria: Ensifera, Caelifera), and Araneae, sampled by sweep-netting between 2008 and 2012. We compiled traits from various literature sources and complemented data from reliable internet sources and the authors’ experience.

## Background & Summary

Arthropod species’ traits are increasingly used in ecology to analyze the consequences of environmental change such as land-use change and intensification and climate warming^1–4^. This is because species respond to environmental changes depending on the traits they possess, and because species’ functions in ecosystems are mediated by their traits^5^. In a review on freshwater ecosystems, the functional trait approach has been highlighted as one of the most promising tools emerging for biomonitoring^6^. Moreover, trait-based analyses have been shown to be useful for providing deeper insights into community assembly^7–9^, for enabling worldwide comparisons e.g., of community responses to environmental drivers across different species assemblages^10^, and for predicting biological invasion^11,12^.

Currently, the availability of trait databases for arthropods is still limited. As a consequence, it is largely unclear which traits are favored or selected against by different environmental drivers such as human land use (response traits) and which traits affect ecosystem functioning (effect traits). Thus, the currently available trait information is likely underestimating the consequences of changes in the trait distribution within an arthropod community for ecosystem functioning^13^. As a consequence, a greater effort is needed to collate and provide relevant trait information.

During the last years, trait databases have been established for some arthropod groups in Europe, e.g., for carabids (carabid.org;^14^), cavity-nesting wasps and bees as well as European bees (Scales project; www.scales-project.net/), European hoverflies (Syrph the Net;^15^), and stream macroinvertebrates^16^. For most groups publicly available trait databases are, however, still missing. More importantly, published standardized trait values across several taxonomic groups which would enable a common trait analysis over several taxa, are very rare in the literature, at least for terrestrial invertebrates (see e.g., ref. 17).

Here, we summarize information on the nine traits body size, dispersal ability, feeding guild, feeding specialization (within herbivores), feeding mode, feeding tissue (within herbivorous suckers), plant part (within herbivorous chewers), endophyllous lifestyle (within herbivores), and vertical stratum use for a total of 1,230 species of Coleoptera, Hemiptera (Heteroptera, Auchenorrhyncha), Orthoptera (Saltatoria: Ensifera, Caelifera), and Araneae. All these species were sampled by sweep-netting on a total of 150 grassland plots across three regions in Germany between 2008 and 2012. We compiled traits from various literature sources (reference books and single publications) and complemented data from internet sources such as the spiders of Europe website (http://www.araneae.unibe.ch) and the carabids.org online database, and the authors’ experiences. The data set supports future trait-based studies in central European grasslands and similar habitats and thus has a high reuse value. It further encourages the collection of the same trait data for species from other habitats (e.g., forests) and regions for which this information is readily available. It also encourages the establishment of additional multi-taxa trait data bases and hopefully provides a framework for the collection of traits in ecosystems or regions where complete taxonomic information is currently developed (e.g., in the tropics).

## Methods

### Site description

The arthropod trait database was established in a project within the Biodiversity Exploratories^18^. The Biodiversity Exploratories cover three regions of Germany: (1) the UNESCO Biosphere Reserve Schorfheide-Chorin in the North-East (52°47′25′′-53°13′26′′ N/13°23′27′′-14°08′53′′ E, about 1,300 km^2^ in size, 3–140 m a.s.l.), (2) the National Park Hainich and its surrounding areas in Central Germany (50°56′14′′-51°22′43′′ N/10°10′24′′-10°46′45′′ E, about 1,300 km^2^, 285–550 m a.s.l.), and (3) the UNESCO Biosphere Reserve Schwäbische Alb in the Swabian Jura in the South-West (48°20′28′′-48°32′02′′ N/9°10′49′′- 09°35′54′′E, about 422 km^2^, 460–860 m a.s.l.). Within each of those three regions, 50 grassland plots of 50 m×50 m size are used as experimental sites. The grasslands are continuously managed by farmers by mowing, and/or grazing and/or fertilization. The overall management intensity covers the typical range of management intensity of the respective region^18,19^.

### Field method

Arthropods were sampled by sweep netting on the 150 grassland plots across the three regions in Germany between 2008 and 2012. In June and August (July and September in Schorfheide-Chorin in 2009) all plots were sampled in all years; in additional months between May to October, samples were taken on a subset of plots (nine plots per region). On each plot and during each sampling period three transects of 50 m along the plot borders were sampled with 20 double sweeps each (one double-sweep is defined as moving the net from the left to the right and back perpendicular to the walking direction). A round sweep net with 30 cm diameter was used. Arthropods were stored in 70% ethanol in the field and sorted into taxonomic groups (Coleoptera, Hemiptera, Orthoptera, Araneae and others) in the laboratory and species were determined by taxonomic experts.

Trait data were collected for all 1,230 species of the above named target taxa sampled in all months between 2008 and 2012.

### Species identification method

All species were identified by taxonomic experts of the respective taxonomic group. Specialists were either one of the authors (MMG: Heteroptera, RA: Auchenorrhyncha, TB: Araneae, FK: Coleoptera) and/or additional specialists mentioned in the acknowledgements. Taxonomy follows the classification of Freude *et al.* incl. Supplement volumes^20^ for Coleoptera, World Spider Catalogue WSC^21^ for Araneae, and the Fauna Europaea data base (www.faunaeur.org;^22^) for the other orders.

### Trait assignment

#### Trait sources

Trait data was collected from different literature sources and completed by the authors’ previously collated databases. Trait data of Coleoptera were based on information given in taxonomic literature^20,23–26^ and trait databases^14^. Trait data of Auchenorrhyncha were based on information in taxonomic literature^27,28^. Trait data of Heteroptera were based on information in taxonomic literature^29^. Trait data of Araneae were based on information in faunistic literature^30–49^, and on information in the online determination key http://www.araneae.unibe.ch ^50^. Trait data of Orthoptera were based on information given in various sources^2,51–59^.

#### Trait collection

We aimed at defining important ecological and morphological traits that can be assigned comparable values across arthropod taxonomic groups. Information for the nine traits body size, dispersal ability, feeding guild, feeding specialization (within herbivores), feeding mode, feeding tissue (within herbivorous suckers), plant part (within herbivorous chewers), endophagous lifestyle (within herbivores), and vertical stratum use was selected and derived from the sources listed above.

#### Trait classification

**Figure 1:**
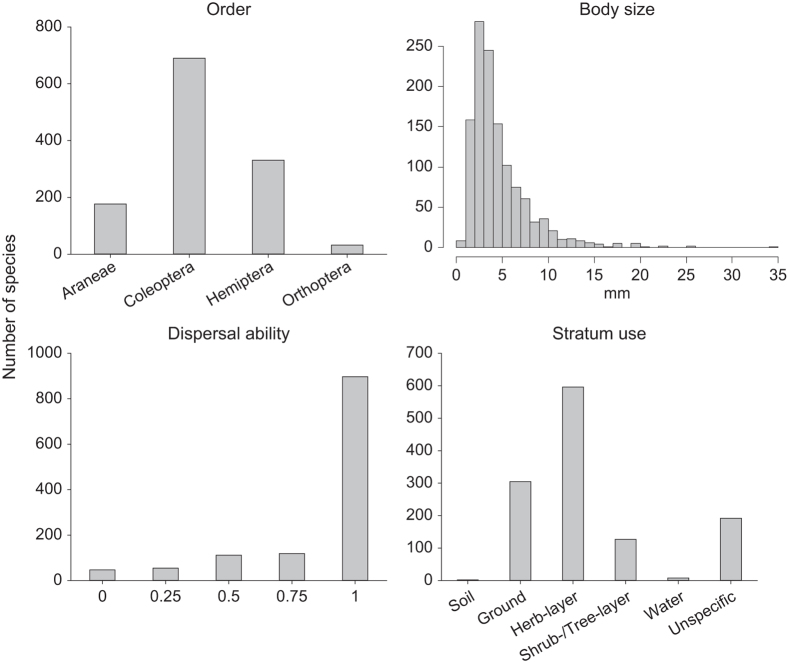
Number of species per order and trait category (body size, dispersal ability, stratum use). Overview on the number of species of the four orders Coleoptera, Hemiptera (Auchenorrhyncha, Heteroptera), Orthoptera (Saltatoria: Ensifera, Caelifera), and Araneae sampled over 5 years in 150 grassland plots (total: 1,230 species). For traits the number of species with each coarse classification or mm-class (body size) is given.

**Figure 2:**
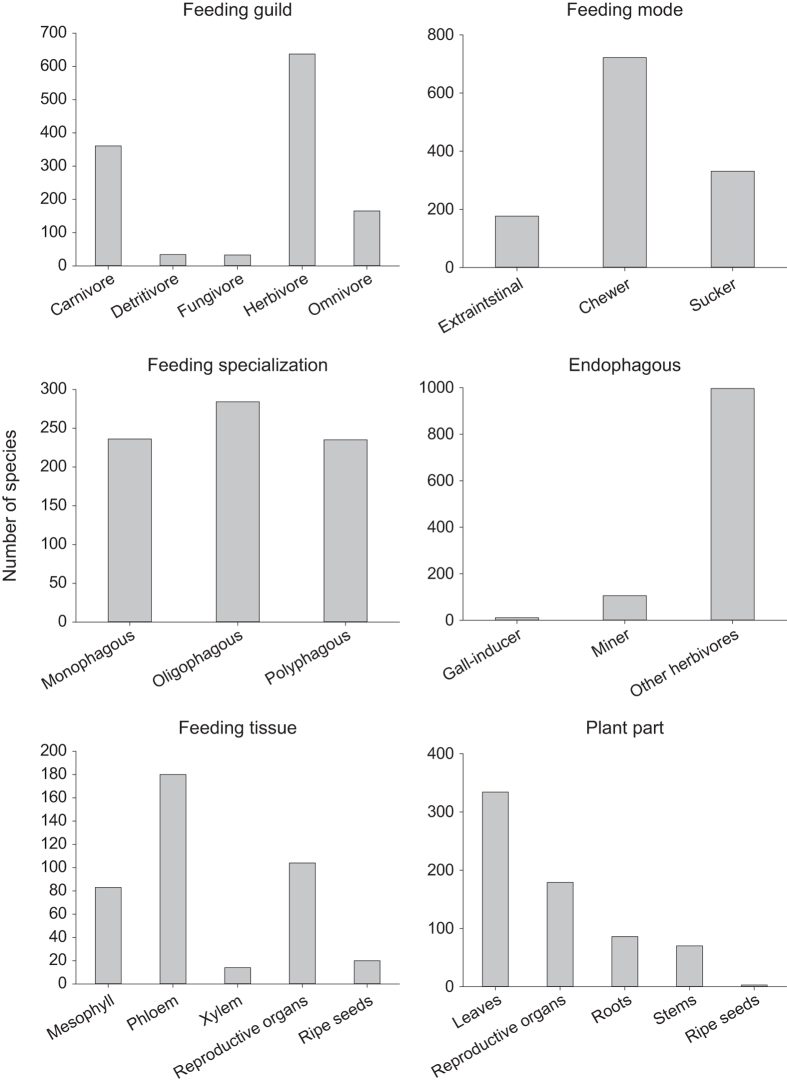
Number of species per trait category, classified according to their feeding ecology. Overview on the number of species of the four orders Coleoptera, Hemiptera (Auchenorrhyncha, Heteroptera), Orthoptera (Saltatoria: Ensifera, Caelifera), and Araneae sampled over 5 years in 150 grassland plots (total: 1,230 species). A more detailed classification is provided for herbivorous species. The figures for feeding mode, feeding specialization, and endophagous lifestyle (note that adults of species with endophagously living larvae might also feed ectophagously) comprise all herbivores. The figure for feeding tissue comprises only sucking herbivores and the figure on feeding plant part comprises only chewing herbivores (note that species might be assigned to more than one category, e.g., many Curculionidae and Elateridae feed on roots as larvae and on leaves as adults).

The traits of the species were classified as follows (see also Figs 1 and 2):

(1) Body size was defined as the mean body length in mm over males and females. We standardized available information within one taxon by using information given in one standard compilation, i.e., Freude *et al.* ^20^ for Coleoptera, Biedermann and Niedringhaus^27^ for Hemiptera: Auchenorrhyncha, Wachmann *et al.* ^29^ for Hemiptera: Heteroptera, and the spiders of Europe website^50^ for Araneae. In Orthoptera, sizes were averaged across values given in different sources^52–54^.

(2) Dispersal ability, ranging from 0 to 1 by steps of 0.25, was defined differently for the groups, depending on available information. For Hemiptera and most Coleoptera, wing dimorphism between males and females was used, assigning 1 for species with fully developed wings in both sexes; 0.75 for predominantly macropterous species; 0.5 for equally brachypterous and macropterous species; 0.25 for predominantly brachypterous species and 0 for always brachypterous species. For other Coleoptera, dispersal ability was based on descriptions of flying ability^20,26^. Species of Araneae were assigned to the five dispersal groups taking into account activity ranges and dispersal strategies (e.g., ballooning and migration), given in different sources^41–48^. Species which are known to be very frequent ‘aeronauts’ and which are observed very frequently outside their main habitat were assigned 1. In contrast, class 0 would include species which never show ballooning behavior, but this did not apply to any sampled species. Species which show ballooning only over a few meter distance and were never observed outside their main habitat were assigned 0.25. When species observations outside the main habitat have been observed more frequently, species were assigned to 0.5 or 0.75 depending on the relative frequency of these observations. For Orthoptera, active dispersal ability is estimated on the basis of the size of the hind wings (alae), the occurrence of macropterous forms and studies of individual movement and colonization dynamics. We followed the classification of Reinhardt *et al.* ^59^ for this trait, which takes these three criteria into account. It is largely based on the (a) wing development of the adults^57^, complemented by (b) results from population studies on individual movements reviewed in^55,56^ and long-term observations of local and regional colonization dynamics reviewed in^55,56^. Additionally, (c) passive dispersal behavior, e.g., by eggs attached to stipes and spread by flooding was considered^51,52^. All flightless species were assigned very low (0) dispersal ability and low (0.25) dispersal ability if they were using passive dispersal behavior. The other species which are able to fly were assigned to the higher dispersal abilities depending on their dispersal behavior considering criteria (b); minor individual movements and colonization dynamics was assigned medium (0.5) dispersal ability, average individual movements and colonization dynamics was assigned high (0.75) dispersal ability and high individual movements and colonization dynamics was assigned very high (1) dispersal ability.

(3) Feeding guild was classified based on the main food during the larval and during the adult stage. For example, many Heteroptera feed on plants in their larval phase but mainly on other arthropods as adults^29^. A coarser (herbivores, predators, fungivores, detritivores, omnivores) as well as a finer classification is given. In the finer classification, all feeding types that substantially contribute to the nutritious source during larval and adult stages of a particular species are given, with less frequent assignments given in brackets. The coarser classification was defined as follows. In Heteroptera, for example, species were classified as herbivores when (a) both nymphs and adults feed exclusively or predominantly on plants, (b) either nymphs or adults feed exclusively or predominantly on plants and plants and arthropods contribute equally to the nutrition of the other stage, or (c) either nymphs or adults feed exclusively on plants and the other stage feeds predominantly on arthropods but uses also plants as nutritious source. All species that use more than one feeding source (plants, animals, fungi, decaying plants or animals) to similar extent across larval and adult stages were classified as omnivores. Classification in Coleoptera is based on Böhme^23,24^, and in Orthoptera on Baur *et al.* ^53^, Detzel^58^, and Maas *et al.* ^52^ All Araneae are carnivore and all sampled Auchenorrhyncha herbivore.

(4) Feeding mode was defined as the way nutrients are ingested. We distinguished between three different modes: extraintestinal digestion, which is common in predacious spiders; chewing of plant or animal tissue by beetles and grasshoppers; sucking on plant or animal sap by Hemiptera.

(5) Feeding specialization was defined as host plant niche breadth of herbivores based on the main higher plant lineages^60^. We classified monophages as species feeding on one plant genus, oligophages as species feeding on one higher plant lineage (i.e., bryophytes, ferns, gymnosperms, angiosperms: monocots, angiosperms: basal eudicots, angiosperms: eurosids, angiosperms: euasterids), and polyphages as species feeding on more than one higher plant lineage. Generally, the classification is based on larval stages. Exceptions are Coleoptera species that develop in dead wood, but feed on plant pollen as adults, in which the specialization of adults was used. Classification is based on Böhme^23,24^, Koch^61^ and Rheinheimer & Hassler^26^ for herbivorous Coleoptera, Nickel^28^ for Auchenorrhyncha, Wachmann *et al.* ^29^ for herbivorous Heteroptera, and Baur *et al.* ^53^, Detzel^58^, Ingrisch & Köhler^55^, and Maas *et al.* ^52^ for herbivorous Orthoptera.

(6) For sucking herbivores we list the feeding tissue on which the species are sucking during the larval and during the adult stage. We distinguished between xylem-, phloem-, and mesophyll-suckers and additionally assigned species that suck on reproduction organs (flowers and unripe seed on the plant), which is common in the Heteroptera families Miridae, Pentatomidae and Rhopalidae and on hard lipid-rich ripe seeds on the ground, which is common in the Heteroptera family Lygaeidae. Classification is based on Nickel^28^ for Auchenorrhyncha and Wachmann *et al.* ^29^ for Heteroptera.

(7) For chewing herbivores we list the plant parts on which the species are feeding on during the larval and during the adult stage. We distinguished between root-feeders, stem-feeders, leaf-feeders and feeders on flowers, pollen and unripe seeds on the plant (combined to feeders of reproductive organs). Classification is based on Böhme^23,24^, Koch^61^ and Rheinheimer & Hassler^26^ for herbivorous Coleoptera, and Baur *et al.* ^53^, Detzel^58^, Ingrisch & Köhler^55^ and Maas *et al.* ^52^ for Orthoptera.

(8) Within herbivorous species we additionally classified species with an endophagous lifestyle of their larvae into gall-inducers and miners following information given in Koch^61^ and Rheinheimer & Hassler^26^ for herbivorous Coleoptera, Nickel^28^ and Nickel & Remane^62^ for Auchenorrhyncha, and Wachmann *et al.* ^29^ for herbivorous Heteroptera. We additionally define the tissue were the larvae are feeding, e.g., leaf or stem miners.

(9) Vertical stratum use was selected as a trait of habitat preference and defined as the main vegetation layer (stratum) in which the species is usually observed as juvenile and adult. A coarser as well as a finer classification is given. We distinguished between soil-dwelling, ground-dwelling, herb-, shrub and/or tree-layer species, and species linked to water bodies. In the finer classification all strata which a particular species uses as habitat are given, with less frequent assignments given in brackets. In the coarser classification, all species that use more than one stratum during their life cycle were categorized as unspecific, thus ignoring different larval and adult habitat niches. For example, several Coleoptera of the family Curculionidae (e.g., genera *Otiorhynchus*, *Phyllobius*, *Sitona*) feed on plant roots in the soil in their larval phase but on leaves in the herb layer as adults^26^. Those species were hence assigned as unspecific in the coarse classification and as soil-and herb-layer species in the finer classification. Where specific information was missing (several Auchenorrhyncha & Coleoptera), the stratum was assigned from the main feeding source or the association with certain plant species. Where information on the species level was missing, information on genera or family level was used, provided that the trait value of the higher taxonomic unit was assumed to be equal for all species based on information given in literature or based on authors’ experience. Classification is based on Platen^49^ and Platen *et al.* ^32,36^, and adapted with information in Maurer & Hänggi^31^, Malten^33,34^, Malten & Blick^35^, Blick^38,39^ and the expert’s experience for Araneae; on Böhme^24^, Freude *et al.* ^20^, Homburg *et al.* ^14^, and Rheinheimer and Hassler^26^ for Coleoptera; on Nickel^28^ for Auchenorrhyncha; on Wachmann *et al.* ^29^ for Heteroptera, and on Köhler^51^, Baur *et al.* ^53^, and Maas *et al.* ^52^ for Orthoptera.

## Data Records

### Data set descriptors

#### Data set identity

Species-level data set for body size, dispersal ability, feeding guild, feeding specialization (within herbivores), feeding mode, feeding tissue (within herbivorous suckers), plant part (within herbivorous chewers), endophagous lifestyle (within herbivores), and vertical stratum use for 1,230 species of Coleoptera, Hemiptera (Heteroptera, Auchenorrhyncha), Orthoptera (Saltatoria: Ensifera, Caelifera), and Araneae.

#### Data set identification code

Our compiled species trait dataset (Table 1) can be found in ArthropodSpeciesTraits.txt (Data Citation 1).

#### Data set description

The data set comprises literature trait data of species that were sampled and measured in a project within the Biodiversity Exploratories^18^ which focuses on the effect of land use on arthropod community composition and related processes (e.g., species interactions such as herbivory or predation) in three regions of Germany^63–65^. For details on the data set see Table 2 (available online only).

### Research origin descriptors

#### Identity

Understanding the consequences of land use in grasslands on trait composition of arthropods.

#### Originators

Martin M. Gossner, Nadja K. Simons, Wolfgang W. Weisser; Terrestrial Ecology Research Group, Department for Ecology and Ecosystem Management, Center for Life and Food Sciences Weihenstephan, Technische Universität München, Hans-Carl-von-Carlowitz-Platz 2, D-85354 Freising, Germany

#### Period of study

2008–2012.

#### Objectives

1. To identify important traits across taxonomic groups, 2. To detect traits that respond to land-use intensification. 3. To analyze the consequences of these trait changes for ecosystem functioning.

## Technical Validation

Field methods followed a standardized protocol. A total of 60×2 sweeps×2 months×5 years=1,200 sweeps per plot is an adequate sampling effort in grasslands^66^. Species identification was cross-checked by different taxonomic experts and at random by barcoding^67^. Trait data was sent out to other experts of species ecology for cross-checks. We compiled traits from various literature sources and complemented data from reliable internet sources and the authors’ experience. If information on traits from different sources varied, experts were asked for their opinion and information of the majority is given. Body-sizes were averaged across indications given in different sources.

## Additional information

Table 2 is only available in the online version of this paper.

**How to cite this article:** Gossner, M. M. *et al.* A summary of eight traits of Coleoptera, Hemiptera, Orthoptera and Araneae, occurring in grasslands in Germany. *Sci. Data*. 2:150013 doi: 10.1038/sdata.2015.13 (2015).

## Supplementary Material



## Figures and Tables

**Table 1:** Detailed account about data-collecting methods and specific data files.

**Sample Name**	**Field method**	**Species identification method**	**Trait assignment**	**Raw Data File**	**Data Repository**	**Data Record Accession (Dryad doi)**
Coleoptera	Sweep-netting	Identification to species level by taxonomic specialists	Trait assignment using existing databases, literature sources and author’s experience	ArthropodSpeciesTraits.txt	Dryad	10.5061/dryad.53ds2
Hemiptera: Heteroptera	Sweep-netting	Identification to species level by taxonomic specialists	Trait assignment using existing databases, literature sources and author’s experience	ArthropodSpeciesTraits.txt	Dryad	10.5061/dryad.53ds2
Hemiptera: Auchenorrhyncha	Sweep-netting	Identification to species level by taxonomic specialists	Trait assignment using existing databases, literature sources and author’s experience	ArthropodSpeciesTraits.txt	Dryad	10.5061/dryad.53ds2
Orthoptera	Sweep-netting	Identification to species level by taxonomic specialists	Trait assignment using existing databases, literature sources and author’s experience	ArthropodSpeciesTraits.txt	Dryad	10.5061/dryad.53ds2
Araneae	Sweep-netting	Identification to species level by taxonomic specialists	Trait assignment using existing databases, literature sources and author’s experience	ArthropodSpeciesTraits.txt	Dryad	10.5061/dryad.53ds2

**Table 2:** Overview of traits defined for all species of Coleoptera, Hemiptera (Auchenorrhyncha, Heteroptera), Orthoptera, and Araneae sampled over 5 years in 150 grassland plots (data set: ‘ArthropodSpeciesTraits.csv’ (Data Citation 1)

**Variables**	**Type**	**Number**	**Levels**	**Abbreviation**	**Description**
Body_size	numeric/metric		Min: 0.31		Mean body length [mm]
			Mean: 4.71		
			Max: 35		
Dispersal_ability	ordinal	5	very low	0	Based on wing dimorphism, flying ability, activity ranges, dispersal strategies, individual movement and colonization dynamics, depending on taxon
			low	0.25	
			medium	0.5	
			high	0.75	
			very high	1	
Feeding_guild	nominal/factor	13	carnivore	c	Fine classification of feeding guild across larval and adult stages; less frequent assignments in brackets
			carni-detritivore	c-d	
			carni-detriti-herbivore	c-d-h	
			carni-fungivore	c-f	
			carni-herbivore	c-h	
			mainly carnivore, rarely herbivore	c-(h)	
			detritivor	d	
			detriti-fungivore	d-f	
			detriti-herbivore	d-h	
			fungivore	f	
			fungi-herbivore	f-h	
			herbivor	h	
			mainly herbivore, rarely carnivore	h-(c)	
Feeding_guild_short	nominal/factor	5	carnivor	c	Coarse classification of feeding guild, indicating main feeding source across larval and adult stages
			herbivor	h	
			detritivor	d	
			fungivor	f	
			omnivor	o	
Feeding_mode	nominal/factor	3	extraintestinal	e	The way nutrients are ingested
			chewing	c	
			sucking	s	
Feeding_specialization	nominal/factor	3	monophagous	m	Host plant specialization in herbivores
			oligophagous	o	
			polyphagous	p	
Feeding_tissue	nominal/factor	13	Mesophyll-cells	m	Fine classification on the plant tissues sucking herbivores are feeding on
			Mesophyll-cells and phloem	m-p	
			Mesophyll-cells, phloem, reproductive organs	m-p-r	
			Mesophyll-cells, phloem, xylem	m-p-x	
			Mesophyll-cells, reproductive organs	m-r	
			Phloem	p	
			Phloem, reproductive organs	p-r	
			Phloem, ripe seeds	p-se	
			reproductive organs	r	
			mainly reproductive organs; rarely mesophyll-cells, phloem	r-(m-p)	
			reproductive organs, ripe seeds	r-se	
			ripe seeds	se	
			Xylem	x	
Feeding_plant_part	nominal/factor	17	Leaves	l	Fine classification on the plant parts chewing herbivores are feeding on
			Leaves, reproductive organs	l-r	
			Leaves, roots	l-ro	
			Leaves, roots, stems	l-ro-s	
			Leaves, reproductive organs, roots	l-r-ro	
			Leaves, reproductive organs, stems	l-r-s	
			Leaves, reproductive organs, ripe seeds	l-r-se	
			Leaves, stems	l-s	
			mainly leaves, stems; rarely roots	l-s-(ro)	
			Leaves, ripe seeds	l-se	
			reproductive organs	r	
			Roots	ro	
			reproductive organs, roots	r-ro	
			reproductive organs, stems	r-s	
			Roots, stems	ro-s	
			Stems	s	
			mainly stems, rarely roots	s-(ro)	
Endophagous_lifestyle	nominal/factor	13	Gall-inducer on leaves	gl	Details on endophagously living larvae
			Gall-inducer on reproductive organs	gr	
			Gall-inducer on stems	gs	
			Leaf-miner	ml	
			Miner in reproductive organs	mr	
			Miner in reproductive organs, stems	mr-ms	
			Miner in roots	mro	
			Miner in roots, stems	mro-ms	
			Miner in stems	ms	
			Miner; mainly stems; rarely roots	ms-(mro)	
			mainly miner in stem; rarely gall-inducer on stems	ms-(gs)	
			Leaf-miner; only during one particular larval stage	(ml)	
			Miner in roots, stems; only during one particular larval stage	(mro-ms)	
Stratum_use	nominal/factor	25	Soil layer	s	Vertical strata used across larval and adult stages; less frequent assignments in brackets
			Soil- and herb layer	s-h	
			Soil- and shrub/tree layer	s-t	
			Ground layer	g	
			mainly ground-, less frequent soil layer	(s)-g	
			mainly ground-, less frequent soil-, herb- and shrub/tree layer	(s)-g-(h)-(t)	
			mainly ground-, less frequent herb layer	g-(h)	
			mainly ground-, less frequent herb- and shrub/tree layer	g-(h)-(t)	
			Ground- and herb layer	g-h	
			mainly ground- and herb-layer, less frequent shrub/tree layer	g-h-(t)	
			Ground- and shrub/tree layer	g-t	
			Herb layer	h	
			mainly herb-, less frequent soil- and ground layer	(s)-(g)-h	
			mainly herb-, less frequent soil-, ground- and shrub/tree layer	(s)-(g)-h-(t)	
			mainly herb-, less frequent ground layer	(g)-h	
			mainly herb-, less frequent ground- and shrub/tree layer	(g)-h-(t)	
			mainly herb-, less frequent shrub/tree layer	h-(t)	
			Herb-, and shrub/tree layer	h-t	
			Shrub/tree layer	t	
			mainly shrub/tree-, less frequent ground- and herb layer	(g)-(h)-t	
			mainly shrub/tree-, less frequent herb layer	(h)-t	
			Water bodies	w	
			Water bodies and herb layer	w-h	
			unspecific—no main stratum	u	
Stratum_use_short	nominal/factor	6	Soil-dweller	s	Main vertical stratum used across larval and adult stages
			Ground-dweller	g	
			Herb layer	h	
			Shrub & tree layer	t	
			water-bound	w	
			unspecific	u	
Remark			non-grassland species	*	Indicates species that do neither obligatory nor facultative occur in grasslands
In the categories feeding guild and stratum a finer classification including all trait levels is given in the columns ‘Feeding_guild’ and ‘Stratum’ of the data table ‘ArthropodSpeciesTraits.csv’. Less frequent assignments are given in brackets. The coarse classification is given in the columns ‘Feeding_guild_short’ and ‘Stratum_short’. Species were assigned unspecific stratum in the coarse classification if they are found equally often in more than one stratum. Species were assigned as omnivores if they equally use more than one feeding source (plants, animals, detritus, fungi). ‘Feeding_specialization’ refers to the host plant breadth of herbivores and includes the levels monophagous, oligophagous, and polyphagous. NA is assigned to all non-herbivores in the data table. ‘Feeding_mode’ refers to the way of ingestion. ‘Feeding_tissue’ refers to the tissue used by sucking herbivores, NA is assigned to non-herbivores and chewing herbivores. ‘Feeding_plant_part’ refers to the part of the plant which chewing herbivores feed on, NA is assigned to non-herbivores and sucking herbivores. ‘Endophagous_lifestyle’ indicates gall-inducing or mining larvae in herbivores, NA is assigned to non-herbivores and herbivores that have no endophagously feeding larvae. The data set also includes data on order, suborder and family as well as the author name and year of species description. For a description of trait assessment and definition see main text.					
